# A Year in Review: Atrial Fibrillation 2024

**DOI:** 10.19102/icrm.2025.16016

**Published:** 2025-01-15

**Authors:** Amier Ahmad, Lydia M. Taranto, Ankur A. Karnik, Rahul Doshi

**Affiliations:** 1 Department of Clinical Cardiac Electrophysiology, HonorHealth, Scottsdale, AZ, USA

**Keywords:** Atrial fibrillation, catheter ablation, pulsed field ablation, stroke

## Introduction

From new ablation technology to strategies to mitigate strokes (and sometimes a combination of both), 2024 was filled with advancements in the management of atrial fibrillation (AF). Here, we will highlight the most important topics covered in the past year.

## Catheter ablation for atrial fibrillation

Pulsed-field ablation (PFA) has dominated this year’s electrophysiology meetings as a breakthrough in the management of AF. Two PFA systems were recently approved by the U.S. Food and Drug Administration (FDA). By using cardio-selective electroporation, PFA offers the theoretical advantage of safety. However, in the Multinational Survey on the Safety of the Postapproval Clinical Use of Pulsed Field Ablation in 17,000+ Patients (MANIFEST-17K), a multinational survey of 116 PFA centers, two PFA-specific complications were noted. In the 17,642 patients undergoing PFA, coronary arterial spasm was noted in 0.14% of patients (25/17,642), while hemolysis-related acute renal failure requiring hemodialysis was observed in 0.03% of patients (5/17,642).^1^ Hemolysis was also noted in a small, non-randomized comparison study of 70 patients undergoing PFA or radiofrequency ablation. An approximately 12-fold increase in red blood cell microparticle concentration was noted in the PFA group, which returned to baseline by 24 h.^2^ The risk of hemolysis correlates with the number of PFA applications and raises concern about non-pulmonary vein targets undergoing ablation.^3^ Additionally, a subgroup analysis from the larger FARAPULSE ADVENT PIVOTAL Trial PFA System vs. SOC Ablation for Paroxysmal Atrial Fibrillation (ADVENT) revealed three subclinical cerebral lesions in the PFA group versus zero in the thermal ablation group.^4^ The most feared complication of AF ablation, atrio-esophageal fistula, has yet to be reported with PFA. This is likely due to the low incidence rate of fistulas and the perceived lack of thermal damage associated with PFA. The latter point remains a topic of debate. A small registry of 43 patients undergoing PFA for symptomatic paroxysmal AF noted a significant temperature rise in the esophagus (>40°C).^5^ The long-term effect of this remains unknown. Like PFA, cryoablation was felt to significantly decrease the risk of atrio-esophageal fistulas when compared to radiofrequency ablation. Recently, Boston Scientific (Marlborough, MA, USA) released a warning regarding a high rate of atrio-esophageal fistulas following cryoablation (7 patients out of approximately 70,000 developed an atrio-esophageal fistula).^6^

The efficacy rates regarding PFA mirror those seen with traditional thermal ablation. Patients included in the Pulsed Field veRsus hIgh-power short-duratiOn Radiofrequency ablatIon (PRIORI) study, a single-center, non-randomized retrospective study, showed similar freedom from atrial arrhythmias between the PFA and high-power, short-duration radiofrequency ablation groups (85% PFA vs. 79% radiofrequency; *P* = .160).^7^ The 1-year results from the prospective, multicenter, single-arm Study for the Treatment of Paroxysmal Atrial Fibrillation by Pulsed Field Ablation System with Irreversible Electroporation (inspIRE) (NCT04524364) of the non–FDA-approved Varipulse system (Biosense Webster, Diamond Bar, CA, USA) in 186 patients with drug-refractory paroxysmal AF cited a similar 76% AF freedom rate.^8^ Overall, these rates are similar to the data reported in the ADVENT trial.^9^ However, there is concern given a high pulmonary vein reconnection rate of 73%. More long-term data are needed to parse out whether the needle shifts in terms of procedural efficacy regarding PFA.

One possible consideration with PFA is the lack of autonomic tone modulation. A sub-analysis from the ADVENT authors compared heart rate variability following PFA versus thermal ablation. Compared to PFA, thermal ablation led to significantly greater increases in heart rate from baseline to 6 and 12 months as measured by Holter monitoring (10 vs. 6 bpm).^10^ Heart rate variability was lower at both 6 and 12 months after thermal ablation. Both parameters are markers of decreased vagal tone, which may be desirable in younger patients with vagally mediated AF.

Efficiency and cost remain a topic of debate regarding PFA. In the ADVENT regulatory trial, the average procedure time was 105 min in experienced United States centers, which is likely on par with experienced operators using thermal ablation.^9^ A real-world study out of the United Kingdom noted shorter procedural times resulting in lower staffing and laboratory costs with PFA, but these savings were offset by substantially greater equipment costs, resulting in higher overall median costs with PFA (£10,010) than with cryoablation (£8106) or radiofrequency ablation (£8949) (*P* < .001).^11^

The type of sedation for AF ablation was also under investigation this past year. In the United States, AF ablations are predominately performed under general anesthesia (GA); however, in resource-limited areas, conscious sedation (CS) is used. Moreover, with the rise of ambulatory surgery centers and a shift to move procedures outside of the hospital, CS is viewed favorably to improve workflow and efficiency. A Danish cohort study compared outcomes in those with first-time thermal AF ablation between 2010 and 2018. Approximately 6400 patients with CS and 1500 with GA were studied.^12^ The primary endpoint was AF recurrence. The GA groups had a higher rate of patients with persistent AF (50% vs. 35%) and those with heart failure (21% vs. 15%). CS was associated with a significantly greater rate of AF recurrence (46% vs. 37%) at 1 and 5 years (67% vs. 63%). The number of re-ablations was higher in the CS group (4% vs. 1.8%). There was no difference in procedural complications. Improved procedural outcomes may be explained by better energy delivery and catheter stability because of limited patient movement and the effect of respirations.

Despite multiple modalities for ablation, the question remains if, and when, to ablate patients. The year 2024 delivered the first sham-controlled trial of AF ablation. The Randomized Sham-controlled Study of Pulmonary Vein Isolation in Symptomatic Atrial Fibrillation (SHAM-PVI) involved two centers in the United Kingdom at which 126 patients with symptomatic AF were randomized to cryoablation or sham procedure.^13^ The sham procedure involved femoral venous access and phrenic nerve pacing. Cardioversion was performed in both groups for the 80% of patients with persistent AF. Patients were predominately men (71%) with an average age of 67 years and an AF history of >2 years. All patients underwent placement of an implantable loop recorder prior to randomization to quantify AF burden. The primary endpoint was AF burden at 6 months, with a secondary endpoint of quality-of-life measures. Unsurprisingly, the change in AF burden from baseline was 60% in the ablation arm and 35% in the placebo arm. All quality-of-life measures were significantly improved in the ablation arm. Although these data demonstrate symptomatic improvement following AF ablation, the question of the placebo effect remains. The trial was enriched with patients most likely to benefit from ablation (highly symptomatic persistent AF patients). A separate control arm that did not undergo true or sham ablation would be needed to truly delineate the placebo effect. Further placebo-controlled trials are underway to help answer this question (NCT05119231, NCT04272762, NCT03907982).

The timing of ablation remains a significant topic of interest. The paradigm of AF posits “AF begets more AF.”^14^ As paroxysmal AF develops into persistent AF, the success rate of an ablation decreases.^15^ This theory has led to the development of a “door to ablation time,” with shorter times being viewed favorably. An observational study within the Catheter Ablation of the Posterior Left Atrium (CAPLA) randomized controlled trial (RCT) aimed to assess this theory. The door-to-ablation time of 334 patients was broken into quartiles (0–12, 13–28, 29–66, >67 months). The authors concluded that shorter door-to-ablation times had lower rates of AF recurrence, but the differences were modest, and all quartiles had a very low AF burden and similar improvements in quality of life.^16^ The study has significant limitations—namely, that it is underpowered, and the initial trial was performed to examine the effect of posterior wall isolation with pulmonary vein isolation. However, the authors show that AF ablation can still be beneficial in those who have had AF on and off for up to 5 years.

## Atrial fibrillation monitoring

We again saw a negative trial for AF screening. ReducinG stroke by screening for UndiAgnosed atRial fibrillation in elderly inDividuals (GUARD AF) was an RCT testing whether AF screening in older adults (>70 years) using a 14-day monitor could identify AF and reduce stroke. The trial was performed at 150 sites in the United States. Approximately 12,000 patients (average age, 75 years; mean CHADS_2_-VASc score, 3.2 points) were enrolled. The median follow-up was 15 months. The observed stroke rates were 0.7% in the screening group and 0.6% in the unscreened group. The bleeding rates were similar at 1.9% in the screening group and 1.1% in the unscreened group.^17^ The screening for AF remains limited, as often screen-detected AF is short in duration and associated with low stroke rates.

The monitoring and detection of AF following stroke was also under review this past year. An observational study examining 370 patients who underwent implantation of an implantable loop recorder following a cryptogenic stroke showed AF detection in 121 patients, similar to the results of the Cryptogenic Stroke and Underlying AF (CRYSTAL AF) study.^18^ Of those 121 patients, approximately half had an AF burden of <0.1%, while the other half had an AF burden of >0.1%.^19^ About 90% of those with detected AF were placed on systemic anticoagulation. The incidence of ischemic stroke recurrence was 4.0% (95% confidence interval [CI], 1.9%–7.3%) in 249 patients without AF detection and 5.8% (95% CI, 2.4%–11.2%) in 121 patients with AF detection (odds ratio [OR], 1.47; 95% CI, 0.54–3.95; *P* = .45). This study is hindered by its retrospective methodology and overall low event rate. However, it does reinforce the need for an RCT evaluating the use of an implantable loop recorder in patients following a cryptogenic stroke. This is especially imperative given the recent AF guideline distinction regarding stroke risks and duration of AF.^20^ As we discussed last year, both the Non-vitamin K antagonist Oral anticoagulants in patients with Atrial High-rate episodes (NOAH-AFNET 6) trial and the Apixaban for the Reduction of Thrombo-embolism in Patients with Device-Detected Subclinical Atrial Fibrillation (ARTESiA) trial raised the question of “what amount of AF warrants anticoagulation?”^21^

The detection of AF following cardiac surgery and the decision to initiate or defer anticoagulation remain a challenge for clinicians. These patients often have several thrombotic risks, and recent surgery raises concerns for bleeding. A meta-analysis of observational data investigated the effect of oral anticoagulation for post–coronary artery bypass graft patients diagnosed with AF.^22^ The study included 28 studies (1.7 million patients) and saw an overall average incidence of AF of 24% (range, 8%–38%). The incidence rates of stroke in-hospital (1.6%), at 1 month (1.0%), and at 1 year (0.6%) were low. There was no association of lower stroke or mortality rates with oral anticoagulation compared to no anticoagulation, but there was a 32% higher rate of bleeding in patients on oral anticoagulation. Although further randomized trials are needed to determine the benefit of anticoagulation in this patient population, these data support an overall low stroke risk in these patients, with perhaps a significant bleeding risk. This of course is unrelated to the role of left atrial appendage clipping in patients undergoing cardiac surgery.^23^

## Stroke mitigation

Following the updated 2023 recommendations regarding left atrial appendage occlusion (LAAO), the popularity of occlusion devices continues to grow.^20^ However, there is some concern regarding the long-term effectiveness of these procedures. In a meta-analysis of 48 studies looking at the incidence and prognosis of residual leaks following percutaneous LAAO, a peri-device leak (PDL) was identified by transesophageal echocardiography in 26% of patients.^24^ For any PDL of >0 mm, >1 mm, >3 mm, and >5 mm, the prevalence ORs for thromboembolism were 1.82 (95% CI, 1.35–2.47), 2.13 (95% CI, 1.04–4.35), 4.14 (95% CI, 2.07–8.27), and 4.44 (95% CI, 2.09–9.43), respectively. These results are sobering regarding the long-term patency rates of these devices. The study also reported a higher rate of PDL assessed by computed tomography, although there was not a significant association with future thromboembolism using this parameter. Despite these findings, combining this approach with patients already undergoing a catheter-based ablation seems obvious in patients who are candidates for both interventions. The Centers for Medicare and Medicaid Services has announced a new diagnosis code for concomitant left atrial appendage closure and cardiac ablation. The Comparison of Anticoagulation with Left Atrial Appendage Closure After AF Ablation (OPTION) trial compared the strategy to implant an LAAO device at the time of AF ablation (concomitant) or shortly after (sequential) versus continuing oral anticoagulation after ablation.^25^ The trial enrolled 1600 patients after AF ablation and assigned them to either continued oral anticoagulants or implantation of a Watchman™ device (Boston Scientific) followed by oral anticoagulant discontinuation after 90 days. On average, patients were 70 years of age, with a mean CHADS_2_-VASc score of 3.5 points. Approximately 40% of patients had concomitant LAAO and AF ablation. The primary safety endpoint of non-procedural major bleeding or clinically relevant non-major bleeding occurred in 8.5% of patients in the device arm versus 18.1% in the anticoagulation group (hazard ratio, 0.44; 95% CI, 0.33–0.59; *P* < .001). The primary efficacy endpoint of stroke, systemic embolism, and death was non-inferior in the device arm (5.3% vs. 5.8 in the anticoagulation arm). The decision to exclude non-procedural bleeding from the primary endpoint is questionable, as the main safety concern with the implantation of an LAAO device is procedural bleeding. The secondary endpoint of major bleeding included procedural bleeding, which was similar between the two groups (3.9% vs. 5.0% for anticoagulation). The overall event rate of stroke/systemic embolism was also low (1.2% vs. 1.3% for anticoagulation) and raises the question of whether investigating a third arm of no device/anticoagulation or antiplatelet monotherapy would be helpful. The authors report a complete occlusion rate of 80% at 12 months, similar to in the previously reported meta-analysis. Overall, more data will be needed to determine whether concomitant procedures are here to stay.

Multiple observational studies have shown an association between AF and cognitive impairment. The Blinded Randomized trial of Anticoagulation to prevent Ischemic stroke and Neurocognitive impairment in Atrial Fibrillation (BRAIN-AF) attempted to determine whether initiating an oral anticoagulant in younger patients with AF would reduce the incidence of cognitive decline.^26^ The results were presented at the 2024 American Heart Association scientific meeting. The study randomized approximately 1200 patients (average age, 53 years) to rivaroxaban or placebo. Cognitive testing was performed yearly and at the final visit. The study was terminated early, with the average follow-up reduced to 3.7 years (vs. 5 years) due to the futility of the anticoagulation approach in reducing the risk of cognitive decline.

Finally, the initiation of anticoagulation following stroke was investigated in the Optimal Timing of Anticoagulation After Acute Ischaemic Stroke with Atrial Fibrillation (OPTIMAS) trial. This was an open-label RCT comparing early versus late initiation of anticoagulation in patients with acute ischemic stroke associated with AF.^27^ Approximately 3600 patients were randomized to early (≤4 days from stroke onset) initiation of any anticoagulant (n = 1824) versus late (7–14 days from stroke onset) initiation of any anticoagulant (n = 1824). The primary outcome of recurrent ischemic stroke, symptomatic intracranial hemorrhage, unclassifiable stroke, or systemic embolism at 90 days was similar between the two groups (3.3% in the early group vs. 3.3 in the delayed group; *P* for non-inferiority = .0003). This study challenges the current dogma of delaying anticoagulation following ischemic stroke over bleeding concerns.

## Conclusion

The year 2024 was another exciting year for AF, with several groundbreaking studies. We look forward to 2025 and all the exciting upcoming research.
